# Ancient origin of animal U-box ubiquitin ligases

**DOI:** 10.1186/1471-2148-10-331

**Published:** 2010-10-27

**Authors:** Ignacio Marín

**Affiliations:** 1 Instituto de Biomedicina de Valencia. Consejo Superior de Investigaciones Científicas (IBV-CSIC). Valencia, Spain

## Abstract

**Background:**

The patterns of emergence and diversification of the families of ubiquitin ligases provide insights about the evolution of the eukaryotic ubiquitination system. U-box ubiquitin ligases (UULs) are proteins characterized by containing a peculiar protein domain known as U box. In this study, the origin of the animal UUL genes is described.

**Results:**

Phylogenetic and structural data indicate that six of the seven main UUL-encoding genes found in humans (*UBE4A*, *UBE4B*, *UIP5*, *PRP19*, *CHIP *and *CYC4*) were already present in the ancestor of all current metazoans and the seventh (*WDSUB1*) is found in placozoans, cnidarians and bilaterians. The fact that only 4 - 5 genes orthologous to the human ones are present in the choanoflagellate *Monosiga brevicollis *suggests that several animal-specific cooptions of the U box to generate new genes occurred. Significantly, *Monosiga *contains five additional UUL genes that are not present in animals. One of them is also present in distantly-related protozoans. Along animal evolution, losses of UUL-encoding genes are rare, except in nematodes, which lack three of them. These general patterns are highly congruent with those found for other two families (RBR, HECT) of ubiquitin ligases.

**Conclusions:**

Finding that the patterns of emergence, diversification and loss of three unrelated families of ubiquitin ligases (RBR, HECT and U-box) are parallel indicates that there are underlying, linage-specific evolutionary forces shaping the complexity of the animal ubiquitin system.

## Background

In eukaryotes, protein ubiquitination is a key biochemical mechanism involved in multiple cellular processes, which range from its main role in the control of protein quality and protein levels, as part of the ubiquitin-proteasome system, to the regulation of gene expression [1-3]. Understanding the groups of proteins that are either involved in ubiquitinating or in regulating the ubiquitination system is therefore highly significant. Among these proteins, ubiquitin ligases (also known as E3s) are particularly interesting. In all eukaryotes, E3s are numerous, structurally diverse and, most relevant from a functional point of view, they provide specificity to the ubiquitination process [3]. In the last years, the problem of characterizing how the groups of ubiquitin ligases have emerged has attracted a significant degree of attention. In particular, my group has contributed to the determination of the evolutionary history and patterns of diversification of two classes of stand-alone ubiquitin ligases: 1) the RBR family, a particular kind of ring finger-containing proteins characterized by having a RING1 - IBR - RING2 domain signature [4-8]; and, 2) the HECT family, which includes all proteins with HECT domains [9]. In humans, these families respectively have 15 and 28 members [5,9]. We also analyzed the proteins of the cullin family, which are essential units of ubiquitin ligase complexes. This family includes 8 different human proteins [10].

In some cases, the origin of these E3s can be traced back to early eukaryotic evolution. For example, three of the twelve subfamilies of the RBR family (called Ariadne, ARA54 and Helicase) [4,7,8] or the three main types of cullin proteins [10] are present in both animals and plants. On the contrary, many other proteins have been found to be of more recent origin, e. g. they are animal-specific. When the analyses are focused on the metazoan lineage, a particular pattern has been found in both the RBR and the HECT families, consisting in two distinct processes: 1) an early diversification, in such a way that animals as different as a placozoan, an anemone and a human have basically the same repertoire, and often a similar number, of ubiquitin ligases of a given family; 2) losses of a substantial number of ubiquitin ligases in a few animal lineages, especially in nematodes and urochordates, and to a lesser extent in insects [7,9].

To further determine the generality of this pattern, I decided to undertake the study of another important type of E3 proteins, the U-box ubiquitin ligases (UULs). UULs were discovered more than ten years ago [11]. They are characterized by having a protein domain, the U box, which is structurally related to the RING finger, typical of many other ubiquitin ligases [12,13]. Evidence for several of these proteins to be biochemically acting as ubiquitin ligases, both initiating and elongating ubiquitin chains, was soon obtained [11,14,15]. There are at least seven UUL-encoding genes in humans, namely the two very similar *UBE4A *and *UBE4B *genes (sometimes respectively called *UFD2b *and *UFD2a*), *CHIP *(also known as *STUB1*), *UIP5 *(a. k. a. *UBOX5*), *PRP19 *(a. k. a. *PRPF19, SNEV*), *CYC4 *(a. k. a. *PPIL2, Cyp-60*) and *WDSUB1 *[11,12,14]. Recently, an eighth gene, *ACT1 *(a. k. a. *TRAF3IP2*) has been described as encoding a ubiquitin ligase that may contain a very divergent U box [16].

Functional information for the roles of the products of these genes is variable, from almost non-existent to extensive. The best-known protein is Chip, which acts both as a cochaperone, together with chaperones such as Hsc70, Hsp70 and Hsp90 [17,18] and as a ubiquitin ligase, alone or as part of complexes that include other E3s, such as the RBR ubiquitin ligase parkin [15,19,20]. Recessive mutations in the *parkin *gene are a well-known cause of familial Parkinson disease, and therefore it is of significant interest that Chip is acting also on the products of several other familial Parkinson disease genes, such as *SNCA *(which encodes α-synuclein; [21,22]) and *LRRK2 *[23,24]. The involvement of Chip in interacting and/or ubiquitinating other proteins involved in neurodegenerative diseases, such as Tau and APP (both implicated in Alzheimer disease; [25-28]), Malin (another ubiquitin ligase, involved in Lafora disease; [29]) and, finally, ataxin-1 and ataxin-3 (associated respectively to spinocerebellar ataxia types 1 and 3; [30,31]) indicates general roles in the nervous system. Consistent with a highly pleiotropic phenotype, *CHIP *null mutant mice show shortened life span, accelerated aging and anomalous oxidative stress and protein quality levels [32].

Much less is known of the functions of the rest of UULs. *PRP19 *encodes a splicing factor required for the activation of the spliceosome in both *Saccharomyces cerevisiae *[33] and mammals [34]. It is part of an evolutionary conserved protein complex known as Nineteen/NTC in yeasts and Prp19/CDC5L in mammalian species [34,35] Although is has been determined to have ubiquitin ligase activity [14], the precise roles linked to that activity are unknown. Yeast, *Drosophila *and mouse *Prp19 *null mutants are lethal [36-38]. Two other UUL-encoding genes, *UBE4A *and *UBE4B*, are also related to a yeast gene, *Ufd2*. This non-essential gene was first characterized as encoding a protein able to elongate polyubiquitin chains (E4 protein) [11]. Evidence for ubiquitin ligase (E3) activity for the mouse Ube4a (Ufd2b) and Ube4b (Ufd2a) proteins was soon obtained [14]. As described above for Chip, the Ube4a and Ube4b mouse proteins have been shown to interact with chaperones, such as, respectively, DnaJc7 and VCP/p97 [25,39]. The substrates of Ube4a or Ube4b in mammals remain largely unknown, although some preliminary information has been already obtained (see e. g. [40,41]). In the nematode *Caenorhabditis elegans*, the products of the orthologs of *UBE4B *and *CHIP*, called respectively *Ufd-2 *and *Chn-1*, form a complex with the chaperone Cdc-48 (homologous to mammalian VCP/p97) and ubiquitinate another chaperone, called Unc-45, which is required for correct muscle formation [42,43]. It has been suggested that the same biochemical process occur in humans, in which VCP/p97 gain-of-function mutations lead to a syndrome leading to myopathy, bone anomalies and, often, dementia [43,44]. Presence of human Ube4a protein has been described in multiple tissues while mouse Ube4b protein was detected in cardiac muscle during development and mostly in the nervous system in adults [39,45,46]. Whether the cardiac muscle phenotype and the results described above for worm and mammalian muscles are related is unknown. Consistent with those data, mice harboring loss-of-function *Ube4b *mutations in homozygosis die before birth, while heterozygotes show multiple nervous system anomalies that worsen with age [46]. Overexpression of mouse *UBE4B *also leads to neurodegeneration in parallel to the generation of ubiquitin inclusions [47]. *CYC4*, originally called *Cyp-60*, was first characterized as encoding a divergent cyclophilin, which was only much later shown to be also a UUL [14,48]. Some cyclophilins work as cochaperones, and in fact Cyc4 proteins has been shown to interact with the chaperone Hsp90 [25]. The biochemical functions of Cyc4 are still poorly understood. None of the two potential biological effects described so far for Cyc4 have been related to the protein acting as a UUL [49,50]. In *C. elegans*, the ortholog of *CYC4*, called *mog-6*, is involved in the switch from spermatogenesis to oogenesis in hermaphrodite worms [51]. The last UUL for which there is some information is Uip5, which has been shown also to interact with the VCP/p97 protein, mentioned above [25]. The roles of *WDSUB1 *are totally unknown.

Research in UUL proteins and UUL-encoding genes in animals would benefit by having them included in an evolutionary framework, which provides three different types of data. First, the patterns of presence/absence of the genes in different species, useful not only to understand the evolution of the genes, but also to determine the best animal models and eventually to better understand the phenotypes found in mutants (e. g. potential redundancies among closely related proteins). Second, the speed of the evolution of a same gene in different species, which may suggest either differences in their evolutionary constraints or new functional roles. Finally, the patterns of evolution of the structures of the proteins, which, if changes are present, may lead also to the generation of novel functional hypothesis, testable in the laboratory. Here, I describe the evolutionary history of all UUL-encoding genes in animals, using when needed external data, most especially from the genome of the closest animal relative sequenced so far, the choanoflagellate *Monosiga brevicollis*. I show that these genes follow the same evolutionary pattern as other E3s: diversification in early animal history followed by simplification in particular lineages. In most groups, these UUL-encoding genes are very conservative, but, as described for other ubiquitin ligase families (see above), nematode species turn out once again to be exceptional, given that they have lost three UUL-encoding genes. The functional implications of these results are discussed.

## Methods

To generate comprehensive databases of UULs, TblastN searches were performed using the sequences of the human U-box proteins as queries against the nr, htgs, gss, est and wgs databases of the National Center for Biotechnology Information (NCBI) [52]. The sequences obtained were aligned using Clustal X 2.07 [53] to determine the regions of highest similarity within each gene (see below). All subsequent searches were focused on these conserved regions, the rest of the sequences were discarded. To obtain the final, exhaustive databases, TblastN searches against the same databases described above, using as queries the conserved regions of orthologous genes of several divergent species, were performed until no additional sequences were found. In that way, all the UUL sequences that either belonged to animal species or to the choanoflagellate *Monosiga brevicollis *present in the databases (*circa *April 2010) were selected. *Monosiga *can be used as a convenient outgroup to establish the genes that were already present when animals emerged, given that choanoflagellates are considered the closest living relatives of animals [54]. From this dataset, only complete or almost complete sequences, which could be aligned along the whole length or at least a large fraction of the conserved regions, were kept for the phylogenetic analyses. However, additional, specific TblastN searches were performed to characterize whether fragments of UULs in basal animal species (such as placozoans or cnidarians) or from lophotrochozoan invertebrates (for which sequence data is limited) existed which could have been missed before. Also, nematode-specific searches were performed at the Nembase4 web page [55,56] to confirm the conclusions of loss of UUL-encoding genes in these organisms. Once all the gene-specific databases were finished, each group of orthologous sequences was aligned using again Clustal X 2.07 and the alignments were manually corrected with GeneDoc 2.7 [57].

While this paper was being revised, the final draft genome sequence of the sponge *Amphimedon queenslandica *was reported [58]. Given the interest in including sequences of this species, several of them unavailable in my original searches, I performed additional TblastN searches against the same NCBI databases indicated above. These searches were finished in September 2010. Seven genes or gene fragments were detected in *A. queenslandica *that were incorporated to generate final alignments.

From those alignments, phylograms were obtained using three different methods of phylogenetic reconstruction. First, Neighbor-joining (NJ) trees were characterized using MEGA 4 [59]. Second, Maximum-parsimony (MP) trees were obtained using PAUP* 4.0, beta 10 version [60]. Finally, maximum-likelihood (ML) trees were obtained using PHYML 3.0 [61]. The parameters used were in general the same detailed in [7]. However, two minor changes were made to refine the analyses: 1) for MP, the maximum number of tied trees was increased from 20 to 100 and the tree-bisection-reconnection algorithm, which is more exhaustive and precise than the subtree pruning-regrafting method used in [7], was chosen; 2) For ML, the improved Le and Gascuel matrix of amino acidic substitutions [62] was used instead of the older Blosum62 matrix. In all the analyses, 1000 bootstrap replicates were performed to establish the reliability of the NJ and MP trees. For ML, which is much more computer intensive, 100 bootstrap replicates were obtained. Finally, structural analyses of UUL proteins were performed with InterProScan [63].

## Results

**Figure 1:**
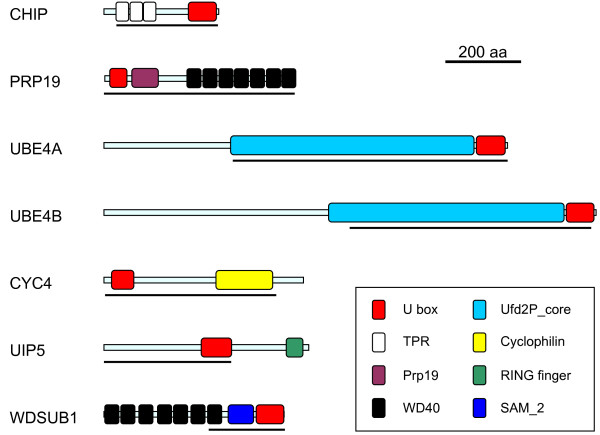
**Structures of human UULs**. Protein domains, according to InterProScan analyses, are indicated. The lines indicate the conserved regions used in the sequence comparison and phylogenetic analyses. These regions would correspond to the following amino acids in the human proteins (in parenthesis, total number of amino acids of the protein; start and end of the conserved region): Chip (303 aa; 25-303), Prp19 (504 aa; 1 - 504), UBE4a (1066 aa; 336-1061), UBE4b (1300 aa; 639-1277), Cyc4 (527 aa; 1-452), UIP5 (541 aa; 1-336), and WDSUB1 (476 aa; 271-472).

Figure 1 shows the structures of the human UULs and details of their conserved regions, all of them including the U boxes. The protein sequences of these conserved regions (detailed in Additional Files 1, 2, 3, 4, 5, 6 and 7) have been used to obtain the alignments on which this study is based. It was fortunate to find out that all the UULs contain long conserved regions of similarity, given that this allows classifying almost all UUL sequences as orthologous of one of the seven human genes. A few exceptions will be discussed below. I will now proceed to detail the results for each of the seven orthology groups, logically naming them as the human genes.

**Figure 2:**
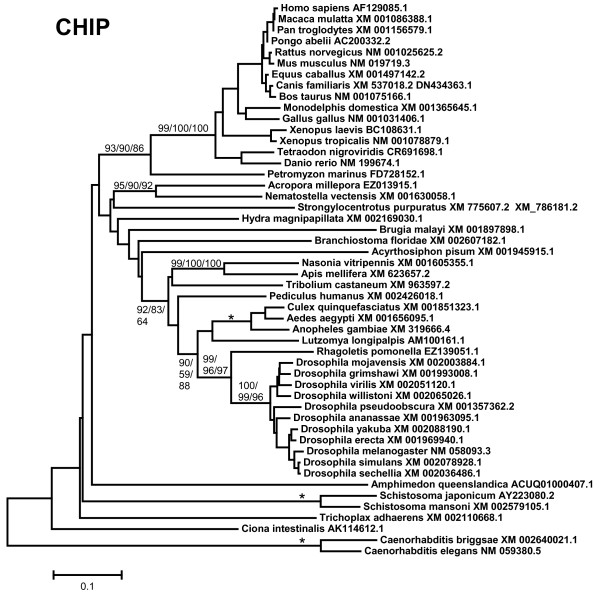
**Chip proteins in animals**. The phylogram indicates the species and accession numbers of the corresponding Chip sequences. Notice the high divergence of the *Caenorhabditis *sequences, discussed in the main text. Bootstrap support, in percentage, is indicated. The numbers respectively correspond to the bootstrap results for the NJ, MP and ML methods of phylogenetic reconstruction (see Material and methods; they are indicated as NJ/MP/ML, around the corresponding branches). In this and the following figures, asterisks indicate 100% bootstrap support according to the three methods. Values have been shown only when at least two of the three percentages were above 50%. Also, for simplification, bootstrap support for external branches (e. g. within mammals or for *Drosophila *species) is not detailed in this or the following trees.

**Figure 3:**
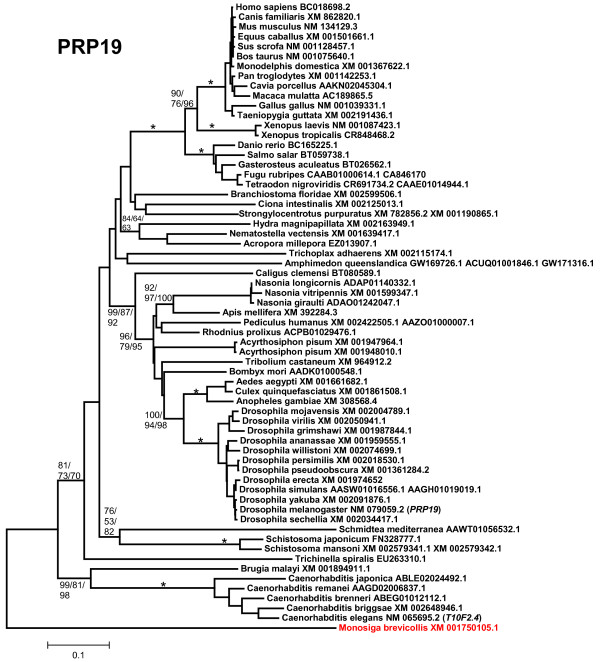
**Phylogram for PRP19 protein sequences**. Conventions as in Figure 2. In red, the sequence of the choanoflagellate *M. brevicollis*. Again, nematode sequences are very divergent.

1) *Chip*: Chip proteins are characterized by containing 3 N-terminal tetratricopeptide repeats (TPRs), involved in protein-protein interactions, and a C-terminal U-box (Figure 1). The gene *Chip *is ancient, being detectable in plants, fungi and multiple protozoan species ([64] and unpublished observations). Figure 2 shows the tree with the complete/almost complete *Chip *sequences detected in animal species. In all species for which genomic data is extensive, a single *Chip *gene is found. Figure 2 includes just two lophotrochozoan species, namely the platyhelminthes *Schistosoma japonicum *and *S. mansoni*. However, fragments of sequences with high similarity with *Chip *were detected in the genomes of many other lophotrochozoans, as molluscs (*Aplysia*, *Euprymna *and *Crassostrea *genera) and annelids (*Capitella*, *Tubifex*, *Hirudo*). Exceptionally, the gene was not found in the choanoflagellate *M. brevicollis*. In the alignments, it was particularly noticeable the high level of sequence divergence in the genes of *Caenorhabditis *nematode species. This is obvious in Figure 2: notice the "normal" position of the closely related nematode *Brugia malayi*, while *Caenorhabditis *sequences are separated from the rest of the tree and have much longer branches. Given that *Drosophila *and *Caenorhabditis *are both ecdysozoans, their divergence from the lineage that gave rise to humans occurred at the same time. Therefore, one would expect to find similar degrees of divergence when the human proteins are compared to either the fly or nematode proteins. However, for Chip that expectation is not fulfilled. Along the conserved region of 279 amino acids analyzed, the resemblance between human and *Drosophila *Chip proteins (identity 56%, similarity 76%) is much higher than the one found in the comparison human Chip vs. nematode Chip (identity: 38%, similarity: 58%).

2) *Prp19*: its origin also predates the emergence of animals, given that it is detectable in many other organisms, such as yeasts, where it was discovered [33] and plants [65,66]. The PRP19 protein is characterized by having an N-terminal U box, followed by a specific region of homology [67] and 7 C-terminal WD40 repeats, also typical protein-protein interaction domains (Figure 1). As shown in Figure 3, single *Prp19 *genes are found in all animal species analyzed and in *Monosiga brevicollis*. Additional partial sequences in molluscs (*Lottia*, *Aplysia*) and annelids (*Platynereis*, *Capitella*), were also detected. As described above for *Chip*, nematode Prp19 protein sequences (this time including also the one in *Brugia*) appear as very divergent in the tree (Figure 3). Again, the resemblance between human and *Drosophila melanogaster *proteins along the conserved region examined for Prp19 (504 amino acids) was substantially higher (65% identity, 81% similarity) than the similitude between human and *Caenorhabditis elegans *(51% identity; 69% similarity).

**Figure 4:**
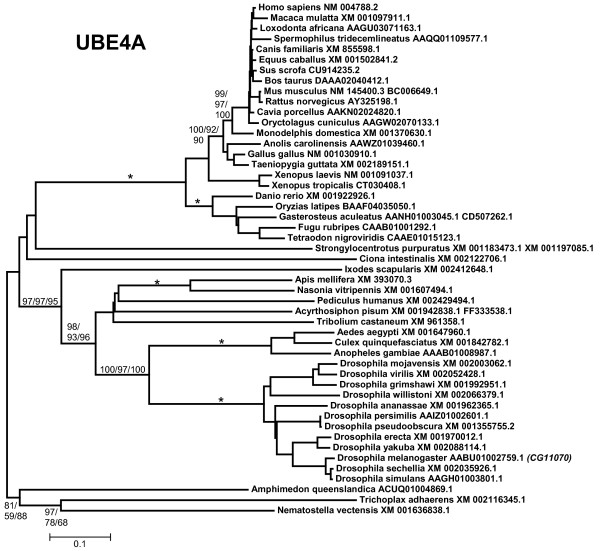
**UBE4A proteins**. Conventions again as in Figure 2. Vertebrates and arthropods form well-supported groups, while the position of the other sequences is ambiguous.

**Figure 5:**
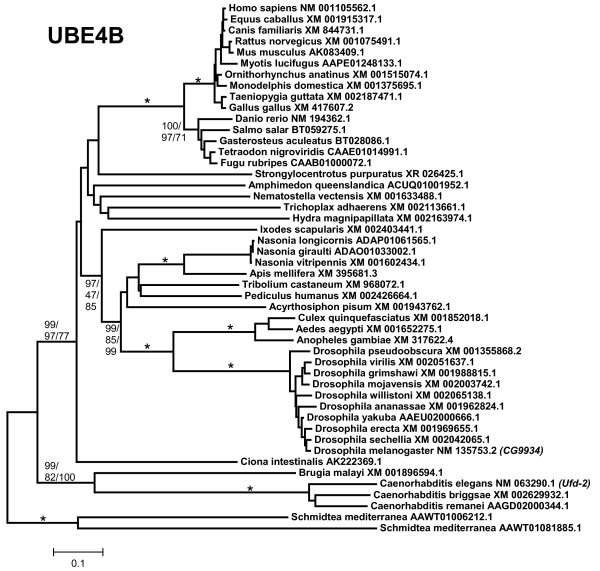
**UBE4B proteins**. As shown in Figures 2 and 3, nematode sequences are again very divergent.

3, 4) *UBE4A *and *UBE4B*: all the proteins related to *Saccharomyces cerevisiae *Ufd2p, among them UBE4A and UBE4B, contain a large stretch of similarity (called "Ufd2P_core" in the Pfam domain database [68]), N-terminally respect to the U box. The U box itself is located at the C terminus (Figure 1). The ancient origin of these genes, present in all eukaryotes, is well documented (e. g. [69]). The duplication *UBE4A*/*UBE4B *seems to have occurred in early animal evolution. Most animals, including sponges, have both genes (Figures 4 and 5). The exception are nematodes (*Caenorhabditis*, *Brugia*), which lack *UBE4A*. Fragments of a single *Ufd2*-like gene were detected in *Monosiga brevicollis*, being thus likely that the *UBE4A/UBE4B *duplication occurred after the choanoflagellate/metazoan split. In addition of those shown in Figures 4 and 5, partial sequences of *UBE4A *and *UBE4B *orthologs were found in molluscs (both genes detected in *Aplysia*, *Lottia *and *Crassostrea*), annelids (both found in *Capitella *and *Hirudo*) and platyhelminthes (*UBE4A *present in *Schistosoma *and *Schmidtea*; notice that Figure 5 shows that *Schmidtea mediterranea *may have two *UBE4B *genes). For *UBE4B*, high divergence of nematode (*Caenorhabditis*, *Brugia*) and also *Ciona *and *Schmidtea *sequences is again suggested by the long branches observed in Figure 5. Along a 638 amino acids-long conserved regions, human UBE4B protein is much more similar to *Drosophila melanogaster *UBE4B (identity 44%, similarity 68%) than to the *Caenorhabditis elegans *orthologous protein (identity 32%, similarity 54%).

**Figure 6:**
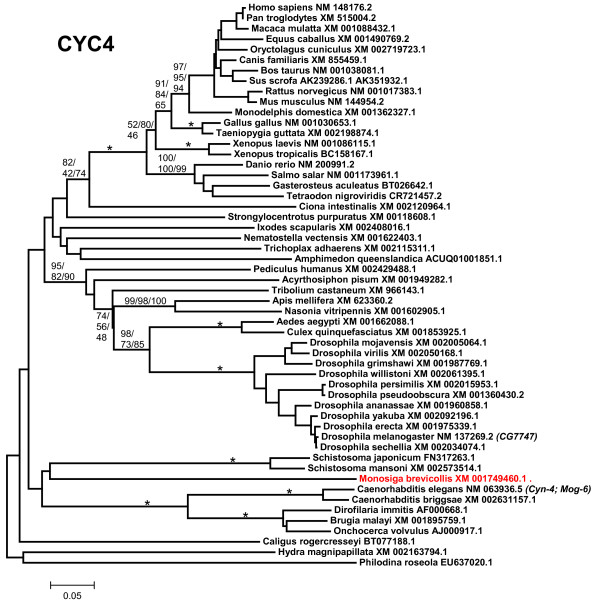
**CYC4 proteins**. *M. brevicollis *branch indicated in red. Notice again the extensive divergence of nematode proteins.

**Figure 7:**
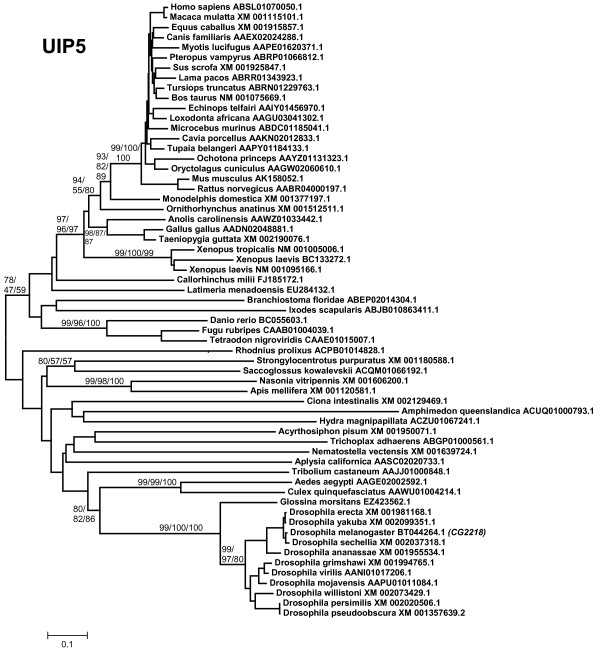
**Phylogram for UIP5 sequences**. In this case, the only well-supported branches include some vertebrates and insects.

**Figure 8:**
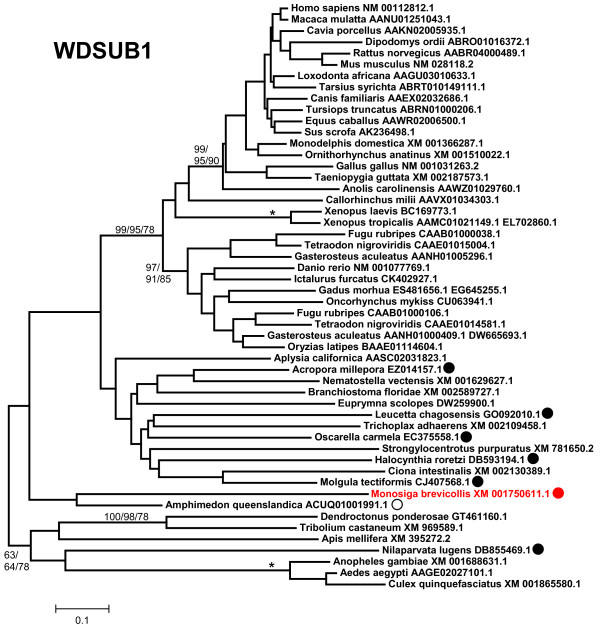
**WDSUB1 proteins**. Dots indicate proteins with structures different from that of the canonical vertebrate WDSUB1 proteins (see main text for details). Black dots: the WD40 domain adjacent to the SAM and U-box domains has not been found. Red dot: 2 SAM domains present but WD40 domains absent. White dot: protein without SAM domain.

5) *CYC4*: CYC4 proteins are characterized by having an N-terminal U box and a cyclophilin (a. k. a. Pro_isomerase) domain near to their C terminus (Figure 1). This type of proteins has been described in fungi [70] and plants [71], so their origin is also ancient. Figure 6 summarizes the phylogenetic analyses for the sequences of all CYC4 proteins detected in animals. Again, a single gene is found in all species, from sponges to humans. It is also present in *M. brevicollis*. Additional fragments of similar sequences were detected in molluscs (*Aplysia*, *Lottia*), annelids (*Alvinella*, *Helobdella*) and platyhelminthes (*Schmidtea*). Notably, once again nematode sequences appear in the tree as highly divergent (Figure 6). This time however, the human/*D. melanogaster *and human/*C. elegans *comparisons do not show a large discrepancy (Hs/Dm: identity 55%, similarity 76%; Hs/Ce: identity 51%, similarity 67%. Length of conserved sequence: 452 amino acids).

6) *UIP5: *The structure of the protein encoded by this gene (Figure 1) is peculiar in two ways. First, the U box is found in all the rest of UULs very close to their N- or C-terminal ends, but here is found in the middle of the protein. Also, UIP5 contains a second domain typical of ubiquitin ligases, the RING finger. This last feature could be interpreted as the U box being dispensable for UIP5 acting as a ubiquitin ligase. However, this is not true. The presence of the U box, but not of the RING finger, is required for the E3 activity of the protein [14]. Also, the U box of UIP5 has been shown to mediate interactions with the ubiquitin-conjugating (E2) enzymes [72]. The evolution of this gene has never been studied before. I have not detected *UIP5 *outside the animals, even *Monosiga *lacks one. It is very unlikely that this is due to distant orthologs being very divergent and thus difficult to detect, given that the C-terminal sequences of UIP5 used to screen the databases are very characteristic, long (336 amino acids, including the U box) and well conserved within animals. Therefore, Figure 7 is a good summary of all the organisms in which *UIP5 *exists. Most animals, including sponges, contain a *UIP5 *gene, but, interestingly, nematodes such as *Caenorhabditis *and *Brugia *have lost it. In lophotrochozoans, *UIP5 *genes have been detected in molluscs (*Aplysia*, *Lottia*), but not in annelids or platyhelminthes. A potential duplicate is observed in the tetraploid amphibian *Xenopus laevis *(Figure 7).

7) *WDSUB1*: the protein encoded by this gene is characterized by containing 7 WD40 repeats and a SAM_2 domain in addition of the U box (Figure 1). SAM domains, as WD40 repeats, are involved in protein-protein interactions. The gene has not been analyzed from an evolutionary point of view in previous studies. I found that *bona fide WDSUB1 *genes are restricted to animals. Figure 8 contains an alignment that has been obtained including in most cases the most C-terminal WD40 repeat (which is highly conserved), the SAM domain and the U box (as schematized in Figure 1). However, there are three exceptions. First, *Monosiga brevicollis *contains a gene clearly related to *WDSUB1 *in sequence (e. g. it has a very similar U box) but not in structure, given that it can be deduced that it encodes a protein with 2 SAM domains but without WD40 repeats (red dot in Figure 8). Second, I found a sequence of the sponge *Amphimedon *that encodes for a protein with 6 WD40 repeats and a U box, very similar to the one in WDSUB1 proteins, but lacks SAM domains (white dot). Finally, a few sequences having just the SAM and U box domains, but lacking the WD40 domain, have been also detected (black dots in Figure 8). Curiously, two of them come from the genomes of species of two other sponges (*Oscarella*, *Leucetta*). Thus, *WDSUB1*-related genes appeared before the emergence of animals, but *bona fide WDSUB1 *genes, encoding proteins with the characteristic WD40 - SAM - U box signature, seem to have originated more recently, after the ancestors of sponges diverged from the ancestor of the rest of animals. Notably, *WDSUB1 *genes are again absent in nematodes (*Caenorhabditis*, *Brugia*) and also in the fruit flies of the *Drosophila *genus, although they are present in some other insect species (Figure 8). They have neither been found in any lophotrochozoan species for which genomic data are available. Therefore, of all the UUL-encoding genes, *WDSUB1 *is clearly the one most prone to be lost. Finally, a duplication has been detected in some fish species (see also Figure 8).

**Figure 9:**
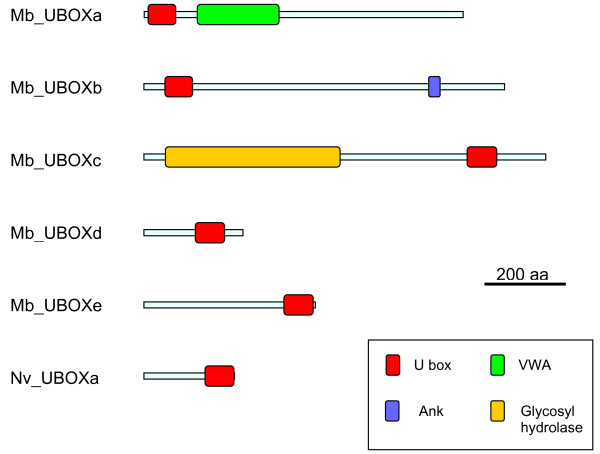
**Structures of the six lineage-specific UULs detected in *Monosiga *and *Nematostella***. Domains were determined with InterProScan, as indicated in the Material and methods section.

As it was indicated in the Introduction, a potential eighth UUL-encoding human gene, *Act1*, has been recently described [16]. Although similarity of the putative U box encoded by *Act1 *with the rest of U boxes is very weak (data not shown), the evolutionary conservation of this gene was also analyzed. It was found to be restricted to mammals. In addition of determining the orthologs of the known human UUL genes, it was also significant to establish whether additional genes existed that were present in other species but not in humans. Along these searches, I detected sequences that correspond to an additional UUL-encoding gene in the cnidarian *Nematostella vectensis *(*Nv_UBOXa*; accession number XM_001634368.1) and surprisingly, five extra genes in the choanoflagellate *Monosiga brevicollis *(*Mb_UBOXa *- *e*; Accession numbers: XM_001744651.1, XM_001750473.1, XM_001748123.1, XM_001749475.1 and XM_001743344.1 respectively). None of these sequences contained the conserved regions characteristic of the seven canonical UUL-encoding genes. The structures of their predicted proteins are detailed in Figure 9. Three of the genes (*Nv_UBOXa*, *Mb_UBOXd *and *Mb_UBOXe*) encode proteins lacking any similarity to other proteins outside the U box domain (Figure 9). More interesting are the proteins encoded by *Mb_UBOXa*, *Mb_UBOXb *and *Mb_UBOXc*, which all have characteristic structures. The first one contains a von Willebrand factor type A (VWA) domain, a protein-protein interaction domain present in a large number of intra- and extracellular proteins [73]. The second contains a single ankyrin repeat, also a well-known protein-protein interaction domain. Finally, *Mb_UBOXc *is predicted to encode a protein with a glycosyl hydrolase domain. Specific searches were performed to detect genes related to these six exceptional ones in other species. In just one case, *Mb_UBOXa*, clearly related sequences were detected in a few protozoans of distantly related groups, such as the heterolobosean *Naegleria gruberi *(Accession number XM_002676557.1), the unclassified anaerobic flagellate *Trimastix pyriformis *(Acc. No. EC839610.1) and the diplomonad *Spironucleus vortens *(Acc. No. GH177899.1) among others. All these sequences potentially encode proteins with the same structure that the one generated by *Mb_UBOXa*. Therefore, although five of the six novel genes discovered in cnidarians or choanoflagellates seem to be lineage-specific, *Mb_UBOXa *is an ancient gene which is not present in animals.

**Figure 10:**
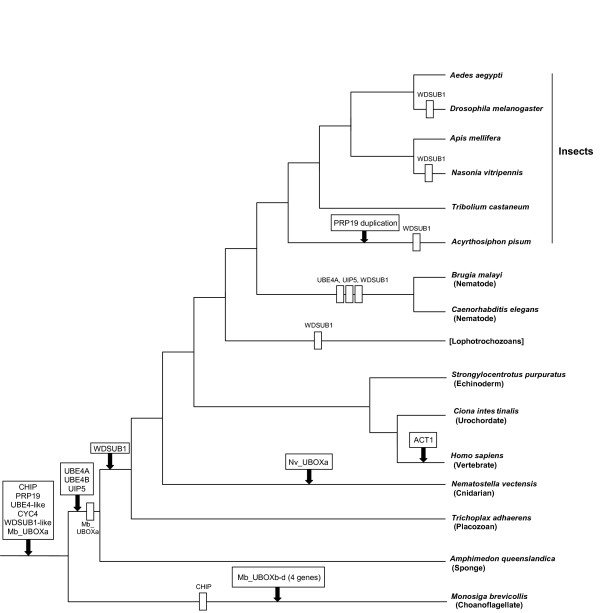
**Patterns of emergence and loss of UULs in selected animals and in *M. brevicollis***. This figure summarizes the most parsimonious hypothesis to explain the patterns of emergence and loss of UUL-encoding genes in selected species. Arrows indicate gene emergences and rectangles, gene losses. *UBE4-like *and *WDSUB1-like *refer to the presence of genes which encode proteins similar in sequence but different in structure to the canonical vertebrate proteins. Lophotrochozoan data are still quite fragmentary and some groups may have lost UUL-encoding genes.

The most parsimonious hypothesis to explain the evolutionary origin of the UUL-encoding genes described in this study is depicted in Figure 10. A rapid summary is that the seven main animal genes (i. e. all but *Act1*) originated long time ago. Proteins structurally identical to the seven corresponding human UULs were already present in early animal evolution, given that they are found in placozoans, cnidarians and bilaterians. Since then, losses have been scarce, although *WDSUB1 *has been lost independently several times (Figure 10). A notable exception to this pattern, also found for RBR and HECT ubiquitin ligases [7,9], is that nematodes lack three UUL-encoding genes (*UBE4A*, *UIP5 *and *WDSUB1*). TblastN searches against the databases at Nembase4, which compiles all the available information for nematode ESTs, confirmed that orthologs of those three genes are not detected in any species. It is also interesting that only a few lineage-specific new genes have been detected. Among all the organisms examined, it turns out that it is the simplest, the unicellular choanoflagellate *Monosiga brevicollis*, the one with the most extensive set of U box ubiquitin ligases. The data so far available suggest that this is largely due to the emergence of additional, novel genes in choanoflagellates.

## Discussion and Conclusions

The evolutionary analysis of the available UUL sequences makes clear the patterns of emergence of the genes that encode these proteins. Four main results have been obtained. The first result is that animal UUL-encoding genes are ancient. Seven of the genes today found in our species appeared before the emergence of animals (*Chip*, *PRP19*, *CYC4*, which are present in many other eukaryotic groups) or in early animal evolution (*UIP5*, a bona fine *WDSUB1 *gene and the duplicates *UBE4A *and *UBE4B*. These last three genes actually derive from related genes already present before animals arose; Figure 10). The only exception is *Act1*, which encodes a protein that may contain a highly divergent U-box and is mammalian-specific. The second result is that the UUL-encoding genes are, as a whole, highly conserved in animals. The only exception is *WDSUB1*, which has been lost independently several times (Figure 10). The third main result is that nematodes have lost three UUL-encoding genes, something that has not occurred in any other lineage. In addition, the remnant nematode genes often have highly divergent sequences, which make them appear in abnormal positions in the phylogenetic trees (see Figures 2, 3, 5 and 6). This high level of divergence has been confirmed by comparing the level of similarity between orthologous *Homo *and *Caenorhabditis *proteins with the level found in the corresponding *Homo*/*Drosophila *comparisons. The expectation is that both levels are identical but, in all cases, the nematode proteins are more divergent, and often much more so (see Results). The final result concerns the unexpectedly large number of UUL-encoding genes found in choanoflagellates but not in animals, a total of five (Figure 9). One of them, *Mb_UBOXa*, turns to be an ancient gene that was lost in early animal evolution.

It is notable how these patterns are often coincidental with those found for RBR and HECT ubiquitin ligase-encoding genes [7,9]. First, most genes of those two families are also either very ancient, being present in most/all eukaryotes, or, alternatively, arose in early animal evolution. In all cases, animals as simple as a placozoan or a cnidarian have sets of genes that are largely the same found in humans. In addition, the structural diversity of RBR and HECT proteins has remained basically the same since animals emerged. A second main result is that losses of RBR and HECT genes in particular lineages, and most especially in nematodes, are also observed. If we put together the results for the three E3 families (RBR, HECT and U-box), it is found that, out of 38 genes present before the cnidarian/bilaterian split, 20 have been lost in *Caenorhabditis elegans*, while only 2 are not present in the anemone *Nematostella vectensis *or in our own species. This result points to an extreme, general streamlining of the ubiquitin system in nematodes. Urochordates, such as *Ciona intestinalis*, are a second group in which both RBR and HECT E3s have been abundantly lost (8 out of 31 genes are missing). However, all UUL-encoding genes are present in *Ciona *(Figure 10), so this pattern is not as general as the one detected in nematodes. Finally, four HECT choanoflagellate-specific genes have been also described [9], suggesting that choanoflagellates have often independently increased their number of ubiquitin ligases. As indicated in the Introduction, several UULs have roles in the mammalian nervous system. However, given their presence in sponges or placozoans, which lack that system, it can be safely concluded that these roles must have been acquired relatively recently respect to the origin of the UUL-encoding genes. However, the fact that both the sponge *Amphimedon *and the placozoan *Trichoplax *have many genes involved in typical neuronal functions [58,74] suggests that many conditions required for the generation of a complex nervous system were already present in the last common ancestor of all animals. In this sense, the early origin of UULs parallels the emergence of other types of proteins which are critical in the function of complex animals, yet appear much earlier in evolutionary history (reviewed by [75-77]).

In conclusion, the analysis of the U-box ubiquitin ligases in animals confirms and extends our previous findings about the general patterns of diversification that explain the current diversity of E3 proteins in animals. An additional result that may have experimental interest is the detection of a general simplification of the ubiquitin ligase set of proteins in a model species such as *C. elegans*. This may facilitate a fast determination in the nematode of the functions of these proteins and perhaps of the ubiquitination system as a whole.

## Supplementary Material

Additional file 1 **CHIP sequences**. Text file containing the aligned sequences in fasta format. Used to generate the phylogram shown in Figure 2. Click here for file

Additional file 2 **PRP19 sequences**. Text file containing the aligned sequences in fasta format. Used to generate Figure 3. Click here for file

Additional file 3 **UBE4A sequences**. Text file containing the aligned sequences in fasta format. Used to generate Figure 4. Click here for file

Additional file 4 **UBE4B sequences**. Text file containing the aligned sequences in fasta format. Used to generate Figure 5. Click here for file

Additional file 5 **CYC4 sequences**. Text file containing the aligned sequences in fasta format. Used to generate Figure 6. Click here for file

Additional file 6 **UIP5 sequences**. Text file containing the aligned sequences in fasta format. Used to generate Figure 7. Click here for file

Additional file 7 **WDSUB1 sequences**. Text file containing the aligned sequences in fasta format. Used to generate Figure 8. Click here for file
